# Polycomb-like 2 regulates PRC2 components to affect proliferation in glioma cells

**DOI:** 10.1007/s11060-020-03538-0

**Published:** 2020-05-21

**Authors:** Fei Wang, Yongying Gao, Ye Lv, Yanwei Wu, Yongzhen Guo, Fang Du, Shixiong Wang, Jiaxiang Yu, Xiangmei Cao, P. Andy Li

**Affiliations:** 1 Department of Pathology, School of Basic Medical Sciences, Ningxia Medical University, Ningxia, 750004 China; 2 Department of Neurology, People’s Hospital of Ningxia Hui Autonomous Region, Ningxia, 750000 China; 3 Department of Oncology, General Hospital of Ningxia Medical University, Ningxia, 750004 China; 4 School of Information Engineering, Ningxia University, Ningxia, 750021 China; 5 Department of Pharmaceutical Sciences, Biomanufacturing Research Institute and Technological Enterprise (BRITE), North Carolina Central University, Durham, NC USA

**Keywords:** PRC2, PCL2, Glioma cells, Proliferation

## Abstract

**Introduction:**

The Polycomb group (PcG) is an important family of transcriptional regulators that controls growth and tumorigenesis. The PcG mainly consists of two complexes, PRC1 and Polycomb Repressive Complex 2 (PRC2). Polycomb-like 2 (PCL2) is known to interact with the PRC2 protein. The role of PCL2 in the development and progression of glioma is unclear.

**Methods:**

We use The Cancer Genome Atlas (TCGA) database to detect the expression of PCL2 in various tumors. 117 cases of clinical glioma (WHOI–IV) were collected, and PCL2 expression and localization were detected by immunohistochemical staining. Glioma cells U87/U251 were infected with overexpressed and interfered PCL2. CCK8 assay, colony formation assay, EdU method, cell cycle and apoptosis were used to detect cell proliferation and apoptosis. Western blot was used to detect the expression of PRC2-related core proteins. After DZNeP intervention, PRC2 protein expression was again measured to discuss the mechanism of PCL2 action.

**Results:**

TCGA database results and immunohistochemical staining results suggest that PCL2 is highly expressed in gliomas. We found that the PCL2 gene promoted tumor cell proliferation, enhanced the colony formation ability, and increased S phase in the cell cycle. The overexpression of PCL2 upregulated the expression levels of EZH2 and EED (two core members of PRC2), decreased the expression of SUZ12, increased the level of H3K27 trimethylation (H3K27me3), H3K4 dimethylation (H3K4me2), and decreased H3K9 dimethylation (H3K9me2). The result after interfering with PCL2 was the opposite.

**Conclusions:**

As an important accessory protein of PRC2, PCL2 can not only change the expression of PRC2 components, but also affect the expression level of Histone methylation. Therefore, PCL2 may be an important hub for regulating the synergy among PRC2 members. This study revealed PCL2 as a new target for tumor research and open up a new avenue for future research in glioma.

**Electronic supplementary material:**

The online version of this article (10.1007/s11060-020-03538-0) contains supplementary material, which is available to authorized users.

## Introduction

Polycomb group (PcG) proteins are conserved epigenetic transcriptional repressors that control numerous developmental gene expressions and have recently been implicated in the modulation of embryonic stem cell (ESC) fate [1]. PcG proteins were first described in D. melanogaster, in which they regulated epigenetic states and proper repression of homeotic genes during development [2, 3]. Most PcG proteins are part of transcriptional repression complexes, termed polycomb repressor complexes (PRCs). Two major PRCs have been identified: PRC1 and Polycomb Repressive Complex 2 (PRC2). PRC2 also contributes to chromatin compaction and catalyzes the methylation of histone H3 at lysine 27 (H3K27me3) [4].

Polycomb-like 2 (PCL2; Metal regulatory transcription factor 2 (MTF2)) is a catalytically inactive polycomb-like (PCL) family protein that has been shown to recruit PRC2 to the loci of target genes in ESCs [1, 5, 6]. In Drosophila, Polycomb-like (PCL) is present in a subset of PRC2 complexes [7, 8]. PCL2 is thought repressing a subset of genes that are known to stabilize the master regulators of pluripotent gene expression, thus regulating the robustness of the pluripotent gene expression program [9]. Related studies have found that the loss of mammalian PCL2 leads to increased self-renewal and delayed differentiation of ESCs [1]. A reduction in PCL2 results in heightened self-renewal characteristics and inefficient differentiation of the three germ layers [1]. Recent studies have reported that leukemia cells are sensitized to chemotherapy induction and exhibited reduced recurrence in acute myeloid leukemia (LAML)-derived xenograft mouse models in response to MTF2 overexpression or to an MDM2 inhibitor that targets signaling pathways [10]. A powerful study has shown that regular heterozygous mutations occur in the independently replicating histone H3 variant (H3F3A). Cases of GBM with H3F3AK27 mutations show high frequency of TP53 mutations, hypomethylation of DNA, midline location and spread of diffuse pontine glioma, and poor prognosis. Mutations in these genes are closely related to the alternate expansions of specific gene expression profiles, leading to the formation of gliomas [11]. We hypothesized that PCL2 might promote the growth of cells in gliomas by altering the effect of PRC2 on histone methylation modifications. In this study, we first detected the expression of PCL2 in tumors through the TCGA database and verified it by immunohistochemistry. And we overexpressed and interfered with the PCL2 gene in glioma U87/U251 cells, and examined the effects of PCL2 gene on cell proliferation, apoptosis, colony formation, and cell cycle. Further, we detected PRC2-related protein levels and histone methylation.

PCL2 has been found to be associated with the core of the PRC2 complex and is highly enriched at many locations of PRC2 enrichment [1]. The PCL protein interacts with PRC2 via EZH2 and, to some extent, through SUZ12 and histones, similar to RbAp46 and RbAp48 [12]. In this study, we first detected the core members of PRC2 (such as EZH2, SUZ12 and EED) and the levels of H3K4me2, H3K9me2 and H3K27me3. We then used EZH2 inhibitor 3-deazaneplanocin A (DZNeP) HCl [13] to interfere with the overexpression of the PCL2 gene. We finally and we examined cell proliferation, clone formation, and the expressions of EZH2, SUZ12 and EED following the drug treatment. These results indicated that PCL2 is an important interacting partner of PRC2 and that the functions of PCL2 are diverse.

## Results

### High expression of PCL2 in glioma

**Fig. 1:**
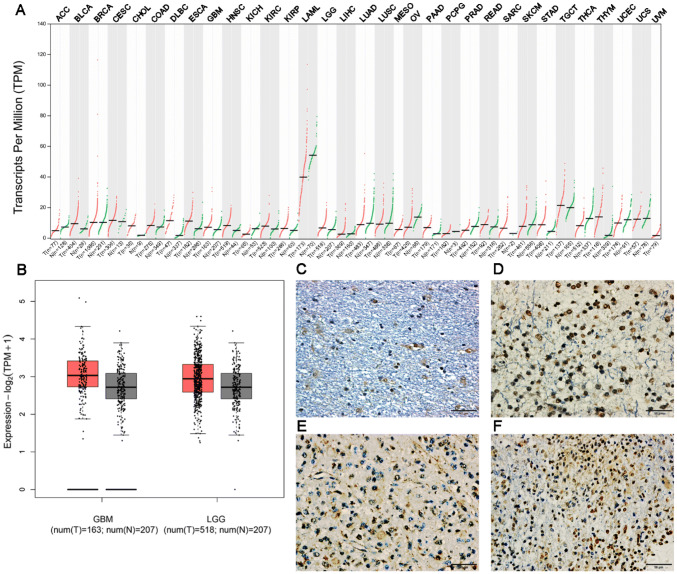
**a** PCL2 expression in 33 tumors from the TCGA database. The red bars represent tumor samples, and the gray bars represent normal tissue samples. **b** PCL2 expression in GBM and LGG. The red box represents tumor samples, and the gray box represents normal tissue samples. **c**, **d**, **e**, and **f** show grade I, grade II, grade III, and grade IV human glioma tissues, respectively (according to the WHO 2016 grading standards). PCL2 expressed dominantly in the nuclei. In one individual, PCL2 expressed in the cytoplasm as well, which appeared as brownish-yellow granules. Bar = 50 μm

We examined PCL2 expression in The Cancer Genome Atlas (TCGA) database. The expression of PCL2 appeared to be different among the 33 tumor types, with lower than normal expressions in adrenocortical carcinoma (ACC), kidney Chromophobe (KICH), LAML, lung adenocarcinoma (LUAD), lung squamous cell carcinoma (LUSC), ovarian serous cystadenocarcinoma (OV), prostate adenocarcinoma (PRAD) and Thyroid carcinoma (THCA), and higher than normal expressions in diffuse large B-cell lymphoma (DLBC), esophageal cancer (ESCA), and head and neck squamous cell carcinoma (HNSC) (Fig. 1a). At the same time, the expression of PCL2 in glioblastoma multiforme (GBM) and brain lower grade glioma (LGG) was also higher than that in the corresponding normal tissues (Fig. 1b). The immunohistochemistry results from human glioma samples were consistent with the results from the TCGA data analysis. PCL2 expression in human glioma tissue samples increased as the grade of the glioma increased (Table 1); the expression was mainly located in the nucleus and was represented by brown-yellowish granules (Fig. 1c, d, e and f). These results suggested that increased PCL2 expression was associated with malignant glioma. Therefore, it is justified to explore the mechanism of the PCL2 gene in the development and progression of glioma.

**Table 1:** Expression pattern of PCL2 in glioma tissue

Tumor grade	Negative	Weakly positive	Positive	Strong positive	Total
LGG (*n* = 42)	8 (19%)	10 (23%)	19 (46%)	5 (12%)	34/42 (81%)
GBM (*n* = 73)	15 (21%)	18 (25%)	33 (45%)	7 (10%)	58/73 (79%)

### Increased expression of the PCL2 gene in U87 cells

**Fig. 2:**
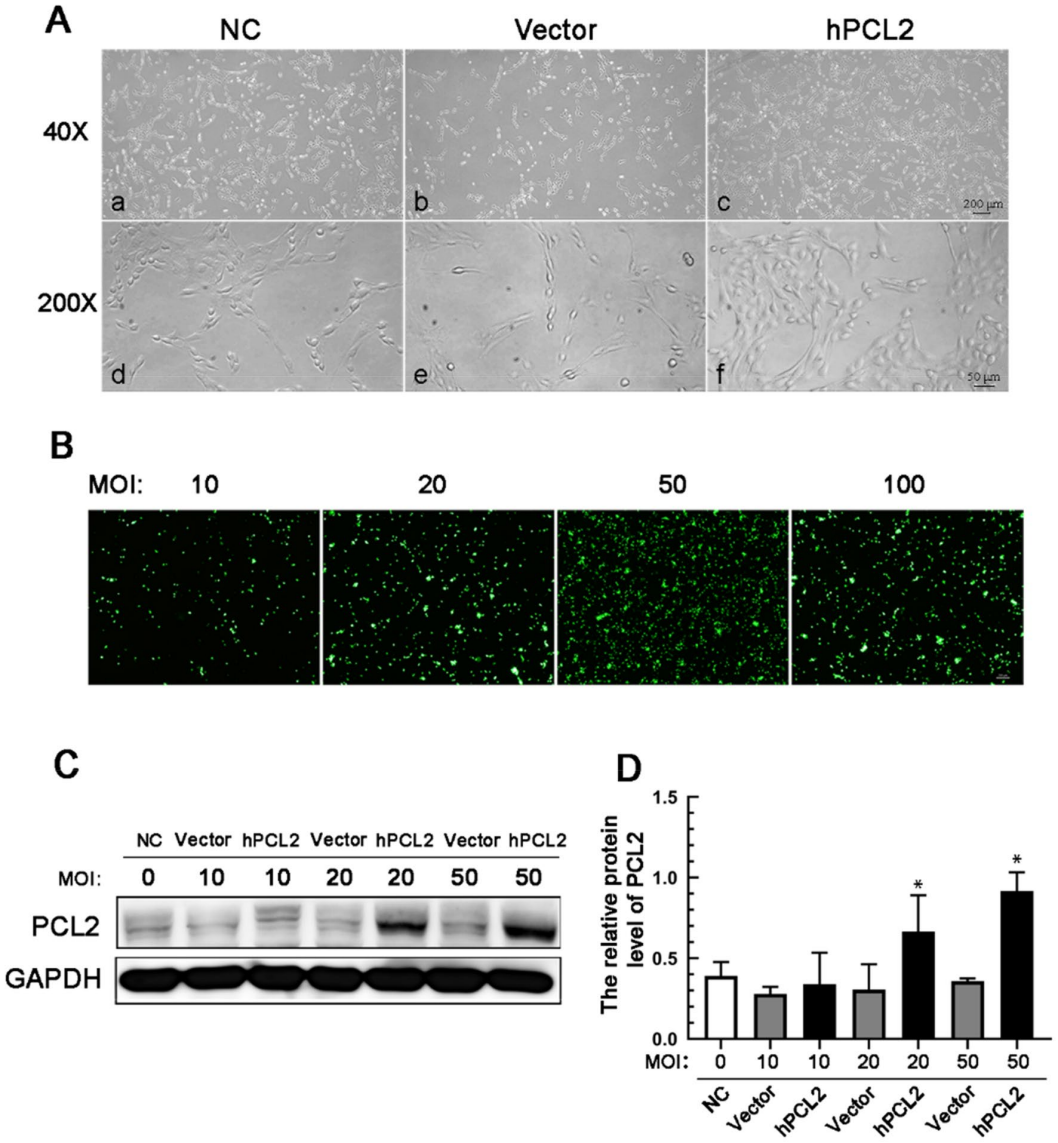
**a** Overexpression of PCL2 with recombinant adenovirus in U87 cells. The cells overexpressing PCL2 after infection with a recombinant adenovirus had a polygonal cell morphology, and the cells were healthy. The experimental concentration of adenovirus did not damage the cells. **b** U87 cells transfected with an adenovirus targeted with GFP probe. **c** and **d** Overexpression of a PCL2 recombinant adenovirus upregulated the expression of PCL2 in U87 cells based on Western blot analysis. Data are presented as mean ± SD of three replicates. *P < 0.05, by one-way ANOVA. Intragroup analysis using the Student–Newman–Keuls (q-SNK) test

To examine the role of PCL2 in U87 cells, we packaged an adenoviral vector expressing hPCL2 and successfully transfected U87 cells with this vector to stably express PCL2; these cells were used for the subsequent assays. The morphology of U87 cells was observed after transfection. The cells were spindle-shaped and were easy to agglomerate. There were no significant differences in the cell morphology among the normal non-transfected control cells (NC), vector-transfected cells (Vector) and high expression PCL2-transfected cells (hPCL2). However, the intercellular space in the hPCL2 group was decreased compared to that in the control group and the empty vector group, and there were more spindle-shaped cells in the hPCL2 group than in the control group and the empty vector group (Fig. 2a). U87 cells infected with adenovirus were observed by inverted fluorescence microscopy at 12 h, 24 h and 48 h. The GFP fluorescence was observed at approximately 24 h, and most obvious at 48 h after infection. The GFP fluorescence was observed in each group of U87 cells, and the fluorescence intensity increased with increases of virus multiplicity of infection (MOI) ratios. As the MOI increased from 20: 1 to 50: 1 and 100: 1, the infection efficiency increased, and the GFP florescence became more stable and persistent (Fig. 2b). Western blotting was used to detect the hPCL2 protein expression. The cells were divided into three groups: the blank control group, the empty vector group and the hPCL2 group, will the cells being infected with the virus at MOIs of 10:1, 20:1, 50:1, and 100:1. The results showed that the PCL2 protein expression level was lower in the blank control group and the empty vector group than in the hPCL2 group. The protein level in the hPCL2 group was significantly higher than that in the control and in Vector groups (Fig. 2c and d). The protein expression level was the highest in the cells that were infected with an MOI of 50:1 (Fig. 2d).

### PCL2 promotes the proliferation of U87 cells

**Fig. 3:**
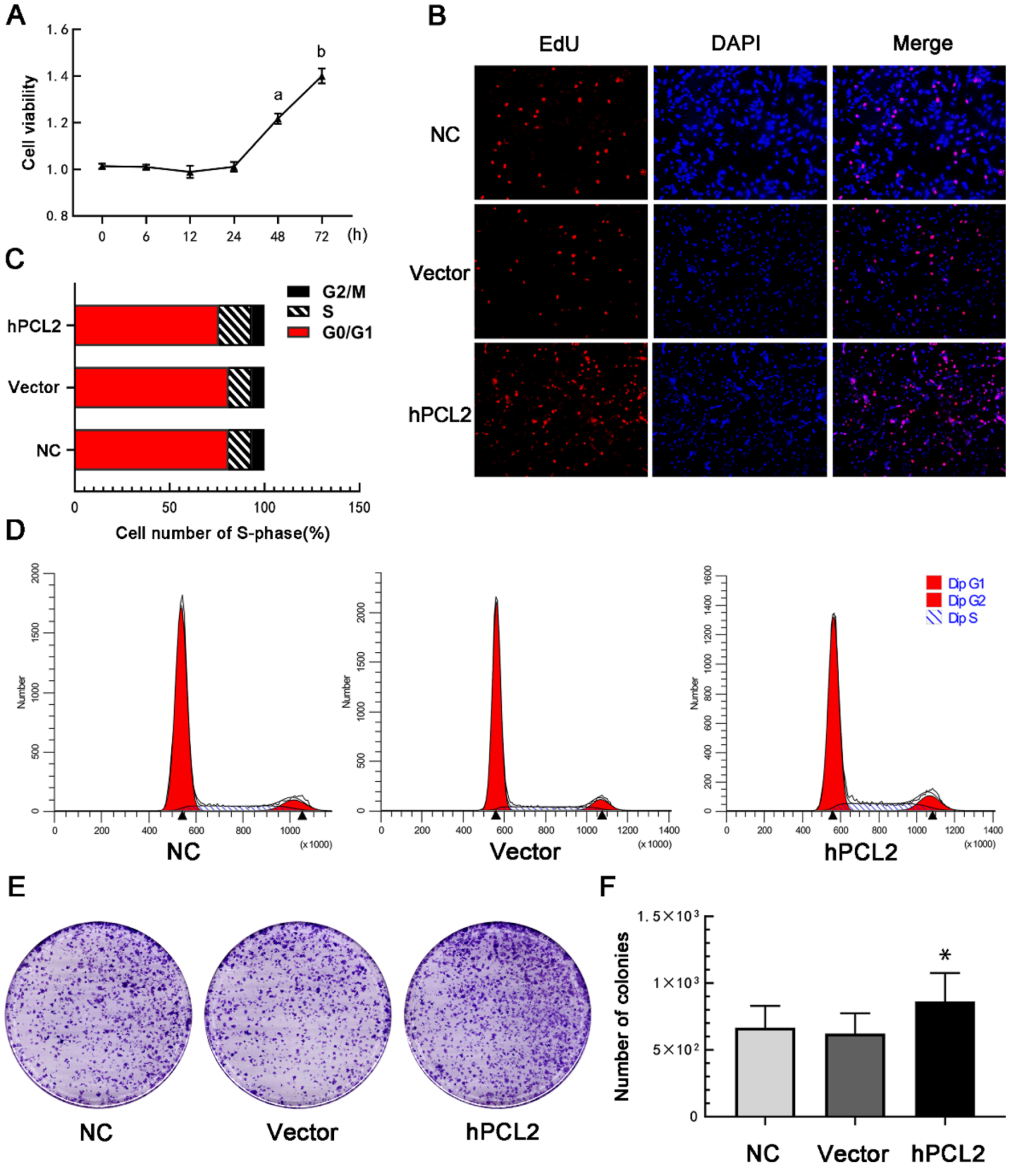
Proliferation ability of U87 cells. **a** The survival rate of U87 cells in the hPCL2 group was significantly higher than that in the blank control group and the empty vector group at 48 h. Data are represented as mean ± SD of three biological replicates. ^ab^*P* < 0.05, by one-way ANOVA. **b** The newly synthesized DNA in U87 cells at 48 h. Red fluorescence indicates the amount of newly synthesized DNA, and blue fluorescence indicates the total number of nuclei stained by DAPI. **c** and **d** The cells in the hPCL2 group had higher proliferative capacity, as shown by the higher number of cells in S phase in the hPLC2 group than in the blank control group and the empty vector group, *P < 0.05 vs the control and vector group. **e** The purple dots represent the colonies that formed. There were more than 50 cells in each colony, as observed under a microscope. **f** Bar chart of with colony numbers and statistics. Data are represented as the mean ± SD of three biological replicates. *P < 0.05, by one-way ANOVA

hPCL2 mainly affected the proliferation of U87 cells. Through a CCK8 experiment, we found that hPCL2 promoted U87 proliferation to some extent. In Fig. 3a, the y-axis indicates the cell survival rate. Compared with the blank control group and the empty vector group, the PCL2 overexpression group exhibited a significantly increased proliferation at 48 and 72 h. This finding was further confirmed using a kFluor555 Click-iT ethynyl deoxyuridine (EdU) imaging assay (Fig. 3b). EdU is an analog of thymidine, which has a methyl group attached to the position-5 C on the deoxythymidine ring by an ethynyl group. EdU replaces the deoxythymidine in newly synthesized DNA during DNA synthesis. EdU-positive cells are proliferative. These results were consistent with our results from cell cycle analysis by a flow cytometer. In our experimental results, U87 cells in the hPCL2 group showed an increase in S phase, which is an important period for the proliferation of cells, while there was no significant difference in the cell cycle in the other groups (Fig. 3c and d). We also observed that compared with the blank control group and the empty vector group, the overexpression group showed a significant increase in the number of U87 cells; additionally, the overexpression group formed compact and deeply stained colonies in the colony formation test (Fig. 3e and f). These data confirm that hPCL2 promotes the proliferation of U87 cells and that the effect of hPCL2 is stable.

### Down-regulation of PCL2 increases apoptosis of U251 cells

**Fig. 4:**
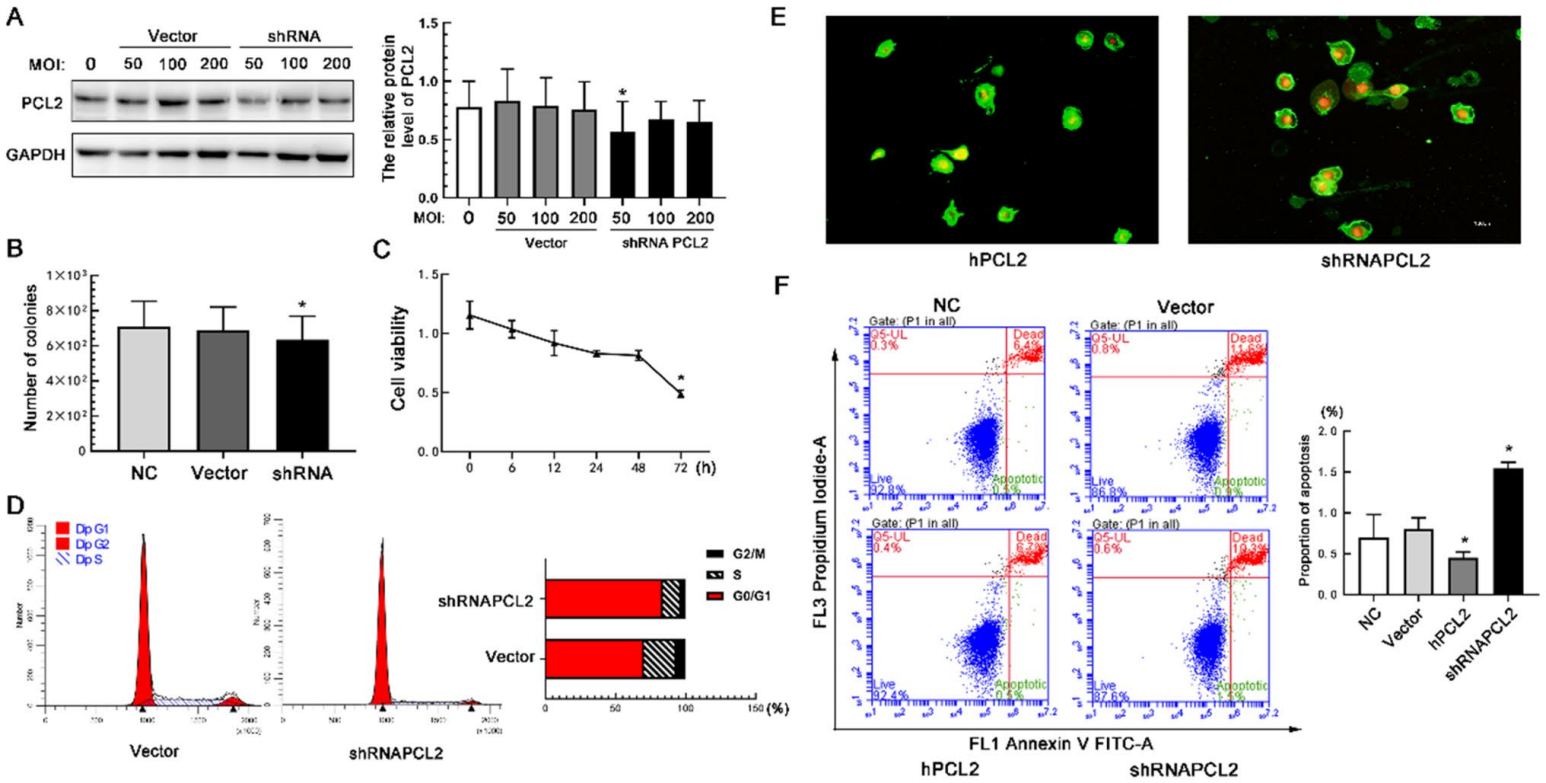
Effect of shRNA PCL2 on U251 cell apoptosis. **a** shRNA PCL2 reduced the expression of PCL2 in U251 cells. *P < 0.05. **b** Reduced number of colonies in shRNA PCL2 cells. *P < 0.05. **c** Cell viability showed a downward trend after shRNA PCL2 transfection. *P < 0.05. **d** The cell cycle of the shRNA PCL2 group was arrested at the G0/G1 phase, and the number of cells entering the S phase was greatly reduced. **e** The apoptosis of the hPCL2 group was different from that of the shRNA PCL2 group. Compared with the hPCL2 group, the cells of the shRNA PCL2 group showed shrinking cell membranes, red staining of the nuclei. **f** Flow chart of apoptosis in blank control group, empty vector group, hPCL2 group and shRNA PCL2 group. shPCL2 increased apoptosis of U251 cells. *P < 0.05

First, we down regulated with the expression of PCL2 in U251 cells using shRNA. The results showed that at the MOI value of shRNA PCL2 50, the interference effect was more obvious (Fig. 4a). Similarly, we performed colony formation experiments and CCK8 proliferation experiments in U251 cells under the condition of PCL2 interference. The experiments confirmed that the number of colonies in shRNA PCL2 group was reduced compared with the blank control group and the empty vector group (Fig. 4b). Cell activity also showed a downward trend an assessed by CCK8 (Fig. 4c). It is obvious that, in the cell cycle, most of the cells were in the pre-replication stage and had not entered the replication stage. The proportion of S-phase cells in the shRNA PCL2 group greatly reduced (Fig. 4d). Through Annexin V-FITC apoptosis detection, we found that the shRNA PCL2 group showed cell membrane shrinkage and the organelles dissolve in spots (Fig. 4e). In addition, apoptosis flow cytometry showed that after down-regulating PCL2, apoptosis of U251 cells increased significantly (Fig. 4f).

### PCL2 affects protein expressions of PRC2 complex core components and histone methylation

**Fig. 5:**
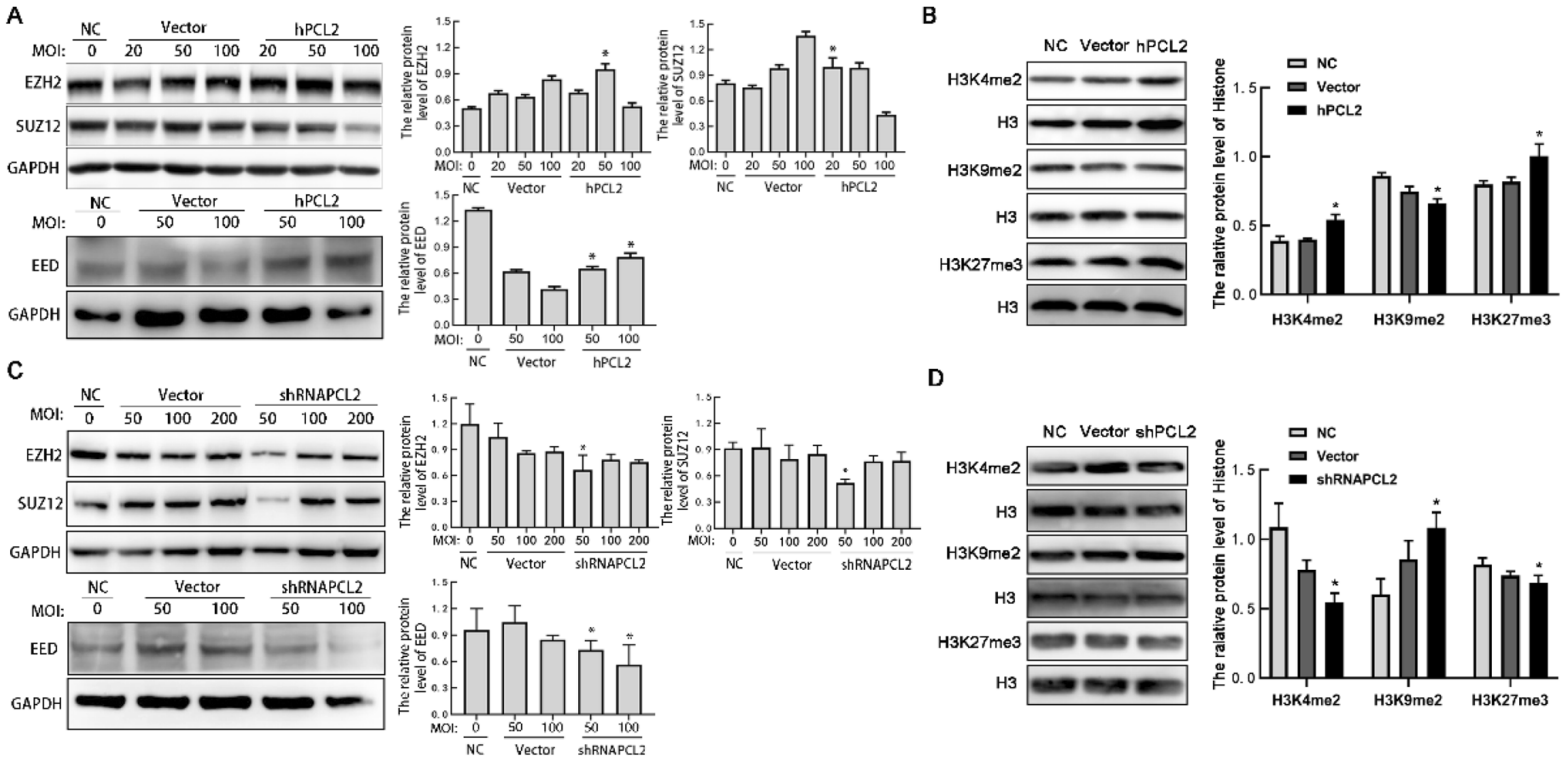
Effect of hPCL2 and shPCL2 on PRC2 complexes and on histone methylation. **a** The expressions of EZH2 and EED were significantly higher in the hPCL2 group than that in the control and the vector group. Among them, the expression of SUZ12 decreased. *P < 0.05. **b** Effect of hPCL2 on the methylation of histone H3K4, H3K9 and H3K29. hPCL2 increased the level of H3K4me2 and H3K27me3, and reduced the levels of H3K9me2. *P < 0.05, one-way ANOVA. **c** Effect of shRNA PCL2 on the protein level of the PRC2 complex core components EZH2, SUZ12 and EED. **d** shRNA PCL2 increased the level of histone H3K9me2 and reduced H3K4me2 and H3K27me3. Data are represented as mean ± SD of three biological replicates. *P < 0.05, by one-way ANOVA

To further study the mechanism of PCL2 in U87/U251 cell proliferation. Western blotting was used to examine the effect of PCL2 expression on the core components of the PRC2 complexes. Compared with the control group, the hPCL2 overexpression group showed moderate increases in EZH2 expression (Fig. 5a) and other components of the PRC2 complex (such as EED). On the contrary, the expression of SUZ12 showed an obviously decline. Next, we examined whether the function of hPCL2 involves modification of histone methylation. We found that hPCL2 increased the levels of H3K4 and H3K27 methylation, but reduced the levels of H3K9 methylation (Fig. 5b). In addition, under the condition of PCL2 interference, we found that its effect on the core protein of PRC2 was opposite to that of hPCL2 in EZH2 and EED, but the change of SUZ12 was not significant in shRNA PCL2 group (Fig. 5c). Further, correspondingly, the effect of shRNA PCL2 on methylation of the corresponding histones was opposite to that of the hPCL2 group (Fig. 5d). These data suggest that cell proliferation may be caused by increased EZH2 expression in response to PCL2, which may affect histone methylation. The functions of PCL2 are diverse, and we thought these functions should be discussed in detail.

### 3-Deazaneplanocin A (DZNeP) inhibits the effect of the PCL2 gene on U87 cells

**Fig. 6:**
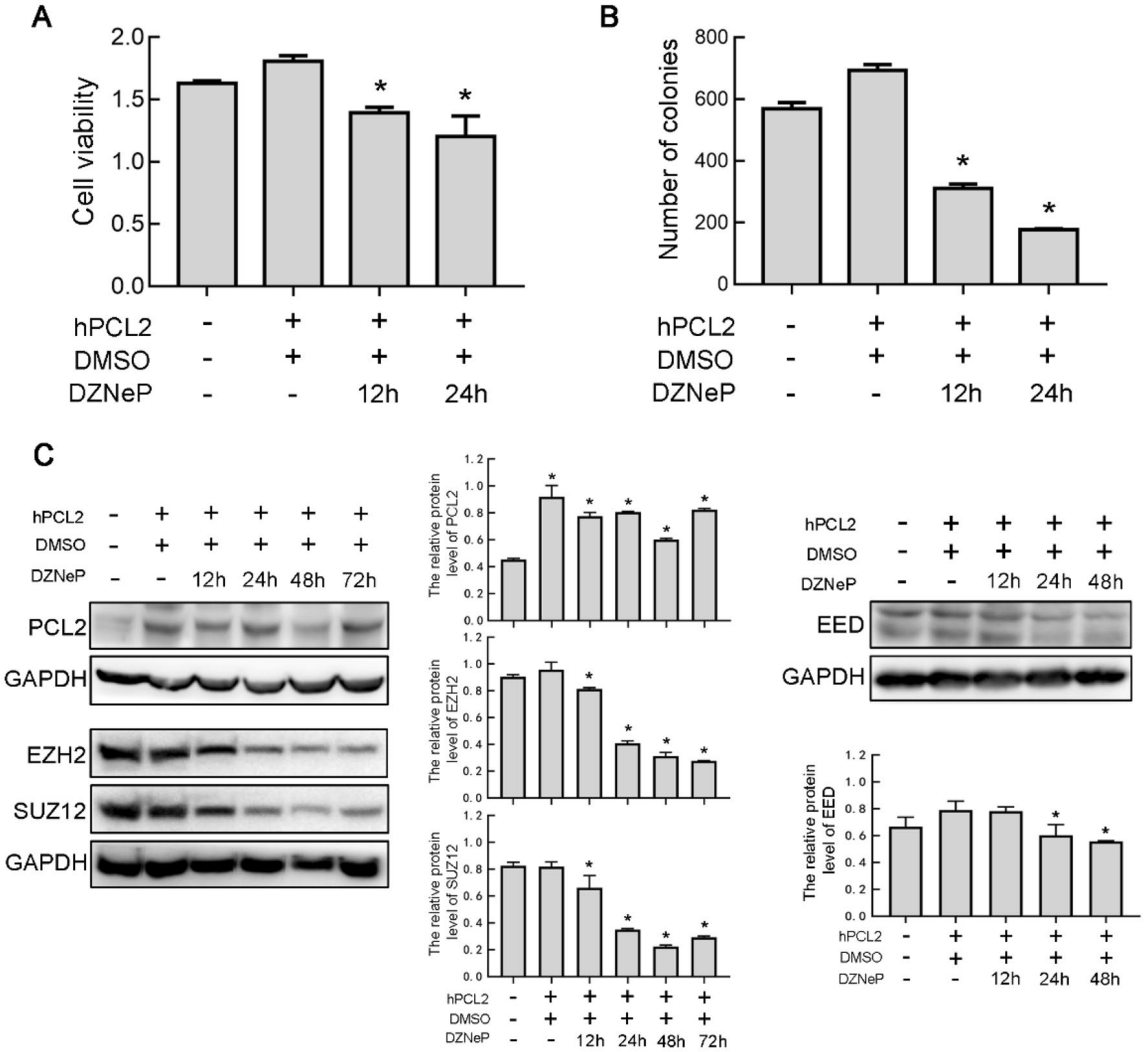
Effect of the EZH2 inhibitor DZNeP on the expression of PCL2. **a** Effects of DZNeP (10 µM) on the survival rate of U87 cells overexpressed hPCL2. **b** The number of new colonies formed in DZNeP-treated cells (10 µM). **c** Effect of DZNeP on the protein levels of the core components of the PRC2 complexes. Data are represented as mean ± SD of three biological replicates. *P < 0.05, by one-way ANOVA

DZNep is an S-adenosylhomocysteine hydrolase inhibitor and a pharmacological inhibitor of histone methylation. The effect of DZNep on cancer cells is relatively specific for targeting EZH2 [13]. According to reports in the literature, the PCL protein interacts with the PRC2 complex through EZH2 [12]. We used the EZH2 inhibitor DZNeP to verify whether the proliferation of PCL2 occurs via EZH2. We found that the inhibitory effect of DZNeP was the greatest after 24 h (Fig. 6a). In the colony formation experiment, we could obviously see that the number of colonies was reduced after DZNeP treatment (Fig. 6b). We demonstrated that in U87 cells, DZNeP reduced the increases in the EZH2 protein level caused by hPCL2 expressions and the protein expression of SUZ12 and EED in PRC2 were reduced compared to that in the control (Fig. 6c).

## Discussion

PcG proteins are known as epigenetic transcriptional repressors that play critical roles in maintaining the cellular memory of fate decisions that are made during development [1]. PcG proteins exist in two main complexes (termed PRC1 and PRC2) and are responsible for the posttranslational modification of the histones H2A and H3. PRC1, which is composed of BMI1, RING1A, RING1B, Cbx and Phc, is responsible for the ubiquitination of histone H2A at lysine 119 [14–16]. PRC2 is composed of a core group of proteins including EZH2, SUZ12 and EED, and it executes its repressive role by modifying chromatin structure via histone methylation [17]. PCLs have been found to be present in a subset of PRC2 complexes [7], and PCL2 is a functional component of PRC2 [18]. PCL1, PCL2 and PCL3 (also known as PHF1, MTF2 and PHF19, respectively) are the three mammalian orthologues of Drosophila PCL [18]. These proteins share protein motifs: a tudor domain, two plant homeodomain (PHD) finger proteins, a PCL extended domain and a carboxy-terminal domain tail [18, 19]. Genome-wide studies have shown that PCL2 co-occupies PRC2 target genes [1, 20]. Various functions have been attributed to PCLs, from the regulation of PRC2 enzymatic activity [12, 21] to the gene recruitment of PRC2 [22]. Previous research found that the knockdown of PCL2 in ESCs resulted in heightened self-renewal characteristics, defects in differentiation and altered patterns of histone methylation [1]. The phenotypes associated with PCL mutations in Drosophila and Xenopus, as well as the colocalization and interaction of PCLs and PRC2, suggest that PCL proteins play a crucial role in PRC2 function [23]. And PCL extended homologous domains are required for efficient recruitment of PRC2 to CpG island-containing promoters in mouse embryonic stem cells [24]. It remains to be determined whether PCL2 could affect tumor progression by acting on the PRC2 complex.

The function of PCL2 gene is complex in tumors. We have examined PCL2 expression in The Cancer Genome Atlas (TCGA) database. The expression of PCL2 in different types of tumor tissue varies greatly, as described in part of Fig. 1. And the expression of PCL2 in GBM and LGG was also higher than that in the corresponding normal tissues. It can also be obtained in immunohistochemical staining experiments that PCL2 is highly expressed in various glioma samples. Our research showed that the hPCL2 obviously promoted U87 cell proliferation, based on CCK8 experiments, colony formation experiments and cell cycle analysis. In response to this, shRNA PCL2 showed the opposite effect. Similarly, we confirmed this phenomenon in primary glioma cells (Supplementary Figs. 1–2).

In Drosophila embryos, PCL forms complexes with PRC2 and maximizes its catalytic activity at Polycomb target genes [9]. In the larval stage, although PCL does not form complexes with PRC2, it mediates pleiohomeotic-dependent PRC2-target binding [25]. These findings imply that PCL plays at least two distinct roles in regulating the expression of Polycomb targets by interacting with different protein complexes and suggests that these interactions depend on the developmental stage or cell type [18]. We have found that the over expression of PCL2 enhanced the expressions of the core component proteins of PRC2, such as EZH2 and EED, and decreased expression of SUZ12. Interestingly, shRNA-mediated inhibition of PRC2 subunit EED, SUZ12, or EZH1/EZH2 causes leukemia cells to stop proliferation and differentiation [26]. However, another view pointed out Eed conditional knockout (Eed (Δ/Δ)) mice will die in the short term due to the rapid decrease of hematopoietic cells. Studies have shown that the absence of EED can lead to abnormal differentiation and functional defects of hematopoietic stem cells (HSPCs) [27].It has been reported in the literature that CRISPR/Cas9-mediated SUZ12 inactivation and mutant JAK3 synergistically drive T cell transformation and T-cell acute lymphoblastic leukemia (T-ALL) development [28]. In contrast, in the study of head and neck squamous cell carcinoma (HNSCC) and non-small cell lung cancer (NSCLC), it was found that shRNA-mediated SUZ12 knock-down significantly inhibited tumor cell proliferation, migration and invasion [29, 30]. Polycomb-mediated gene silencing is thought to rely mostly on the regulation of chromatin structure, in part through post-translational modification (PTM) of histones. Among them, there are many studies related to H3K27me3 enrichment and gene silencing [31]. The PRC2 complex is responsible for the methylation (di- and tri-) of Lys 27 of histone H3 (H3K27me2/3) through its enzymatic subunits EZH2. And PCL2 interacts with PRC2 through EZH2, and to some extent through SUZ12 and histone chaperones RbAp46 and RbAp48, further affecting histone modification [12]. Knockdown of PCL2 disrupts global H3K27me3 during differentiation in ESCs [1]. In histone modification, the gene expression of the corresponding site is regulated and the chromatin structure is maintained. Markers at different positions can determine whether the gene is activated or inhibited. H3K9 and H3K27 methylation are related to gene silencing, while H3K4 methylation can activate genes [32]. In our current research, we found that PCL2 gene expression up-regulated H3K4me2 and H3K27me3 and down-regulated H3K9me2. This is probably due to PCL2′s ability to upregulate EZH2. EZH2 is a histone methyltransferase (HMTase) and is able to catalyze the H3K27me2/3. PRC2-EZH2 regulates cellular H3K27me2/3 levels through its EZH2-mediated methyltransferase activity [23]. It has been suggested in the latest research that the mutant H3K27, which is a lethal subunit of glioma, appears in the normal H3. The ability, recruiting target genes on chromatin by PRC2, does not seem to be affected by the H3K27 mutation, but the transcription will be restricted if the deposition of H3K27me3 and me2 in the whole genome is depleted, and results in affecting gene expression of regulating neurogenesis. Removal of the H3K27 mutation can restore H3K27me2/me3 proliferation, impair cell proliferation, and completely eliminate its ability to form tumors in mice [33]. Our research showed that the PCL2 gene up-regulated H3K4me2 and down-regulated H3K9me2, which does not seem to explain that these two changes are directly related to cell proliferation. But studies have shown that in specific cell cycles, the modification of H3K4me2 and H3K9me2 is related to chromatin inhibition [34]. Therefore, the mechanism that PCL2 alters the methylation of histone sites and the changes in cell fate caused by changes in these histone sites will be our future investigation.

EZH2 possesses many domains and acts as a platform for interaction between EED and SUZ12, thus promoting the formation of active enzymes. The N-terminal region of EZH2 forms a tight band around the EED, which enhances the interaction [35, 36]. Therefore, enzymatic action by EZH2 at target genes requires the binding of SUZ12 and EED [9, 37]. DZNeP blocks EZH2-associated H3K27me3 and reactivates PRC2-silenced genes to induce apoptosis and to amplify the DNA damage response (DDR) and the cytotoxic effects of chemotherapy in malignant cells but not in normal cells [4]. We used DZNeP to inhibit EZH2 and then observed the regulation of the core members of PRC2 by PCL2. We found that the expression of PCL2 increased the protein level of EZH2, while DZNeP inhibited the expression of EZH2. At the same time, the number of new colonies decreased with DZNeP treatment. The application of the EZH2 inhibitor DZNeP could not completely alleviate the impact of PCL2 on the core components of PRC2. Therefore, we speculate that PCL2 does not only act on genes through EZH2, and there may be other downstream genes that play a direct role in the activity of PCL2. The core members of PRC2 are mediated by EZH2. In our results, SUZ12 expression decreased in response to the hPCL2, but lowering EZH2 with DZNeP also reduced SUZ12 expression, suggesting that in our experiments, the hPCL2 promoted SUZ12 reduction. However, this effect was independent of the interaction between PCL2 and EZH2. The overexpression of PCL2 not only altered the expression levels of the PRC2 components but also affected histone methylation. Therefore, we speculate that PCL2 may be an important hub for regulating various members of the cooperative PRC2 complex.

## Materials and methods

### TCGA database analysis

The PCL2 expression data of all tumors in this study were derived from the TCGA database (https://portal.gdc.cancer.gov/) and the online analysis software GEPIA2 (https://gepia2.cancer-pku.cn/) #index).

### Patient tissue sample sources and immunohistochemistry

Immunohistochemical staining samples were obtained from the paraffin tissue sections of 115 cases of glioma from the Department of Pathology, Ningxia Medical University. The use of samples in this study was approved by the Institutional Research Board (IRB), and all subjects had been previously provided informed consent. The results of immunohistochemical staining were determined by a semiquantitative method based on the staining intensity (a positive result was indicated by brownish-yellow particles) and the percentage of stained cells. Briefly, for immunohistochemical staining, after dewaxing with xylene and ethanol, an EDTA antigen retrieval solution (pH 8.0) (ZSGB-BIO, Beijing, China) was used for antigen retrieval. Endogenous peroxidase activity was blocked with 3% H2O2. Goat serum was used for blocking at room temperature for 30 min, and the sections were incubated with the primary antibody (PCL2, proteintech, USA) at 4 °C overnight. The next day, after rewarming at room temperature, a polymer enhancer was added and was incubated in a 37 °C incubator for 20 min. After washing with PBS, the secondary antibody (goat anti-mouse IgG, ZSGB-BIO, Beijing, China) was added and incubated in a 37 °C incubator for 1 h in PBS. After washing, DAB (ZSGB-BIO, Beijing, China) was added to stain the slides. Finally, hematoxylin (ZSGB-BIO, Beijing, China) was used to stain the nucleus after dehydration, the slides were sealed, observed under a microscope and images were taken for analysis.

### Cell culture of human glioma U87/U251 cells and PCL2 adenovirus infection

U87 or U251 cells were seeded in dish with DMEM (HyClone, American) containing 10% FBS (HyClone, American) at a density of 2 × 10^5^ cells/100-mm culture dish. The cells were cultured in an incubator with 5% CO_2_ and temperature at 37 °C. Adenovirus infection was performed after the cells had attached for 6–8 h. The titer gradients of the hPCL2 adenovirus and control virus were set to an MOI of 10:1, 20:1, 50:1, and 100:1, and the gradient medium was mixed with 2% FBS in DMEM for the transfection of U87 cells. Because the basal expression of PCL2 is particularly low in U87 cells, they are good for overexpression but not for shRNA knockdown. For this reason, we transfected shRNA PCL2 adenovirus to U251 cells with the MOI of 50:1, 100:1, 200:1.

### Total protein and histone extraction

Adenovirus-transfected cells were trypsinized and centrifuged to obtain cell pellets. An appropriate amount of cell lysate was added into the cell pellet after discarding the medium mixture. We then follow the steps of Total Protein (KeyGEN Biotech, Nanjing, China) and Histone Extraction Kit (Epigentek, USA) to extract the protein-containing liquid supernatant. Next, the total protein and histone protein were quantified in accordance with the requirements of the BSA protein quantification kit (KeyGEN Biotech, Nanjing, China). According to the standard curve, we calculated the value of required total protein concentration was 40 µg/10 µl and the histone concentration was 5 µg/10 µl. After aliquoting, protein denaturation was performed at 95 °C for 15 min, and then we stored the protein at − 20 °C.

### Western blot analysis

Equal amounts of total proteins and histones were separated by SDS-PAGE gel electrophoresis transferred to PVDF membranes (Millipore, USA), and blocked in 10% skim milk for 1 h. The primary antibody diluted by 3% BSA was hybridized to the membrane overnight at 4 °C. The membrane was incubated with the secondary antibody for 1 h at room temperature followed by chemiluminescence using ECL (Thermo Scientific USA) reagent. We then saved the image result and calculate its gray value. The primary antibodies used in the experiment included PCL2 (Abcam, UK), EZH2, SUZ12, EED, H3K4me2, H3K7me2, H3K27me3, H3 (Cell Signaling Technology) and GAPDH (ZSGB-BIO, Beijing, China).

### CCK8 cell proliferation assay

Cells were passaged at a density of 2000 cells/well and seeded in 96-well plates (each well contained 100 μl of medium with 10% serum). After 6 h in culture, the cells were transfected with virus. Transfection times were 6, 12, 24, 48, and 72 h, respectively. After the transfection, the culture medium in each well was discarded. A mixed solution of 100 µl of normal medium and 10 µl of CCK8 (Cell Counting Kit-8, Dongren Chemical Technology, Japan) was added to each well, cultured in a 37° C 5% CO_2_ incubator for 4 h, and measured for absorbance at 450 nm using a microplate reader (Thermo Scientific, USA).

### Colony formation experiment

1000 cells were seeded into a 100 mm petri dish, and the cells were transfected with the corresponding viral vectors according to different groups. After 10 days, cells were aggregated under a microscope observation. The cells were fixated with 4% paraformaldehyde (Leigen Biotechnology Co., Ltd. Beijing, China) at room temperature for 20 min after discarding culture medium. A total of 4 ml of crystal violet solution was added into each dish, and the cells were stained for 30 min at room temperature and then washed with water. After drying at room temperature, we took pictures, counted the number of colonies with Image-Pro Plus 6.0, and then performed statistical analysis.

### Cell cycle and apoptosis detection

Cell cycle detection, a total of 1 × 10^6^ cells collected and were washed twice with cold PBS. Seventy-five percent frozen ethanol was used to fix the cells overnight at 4 °C, and then the cells were washed again with cold PBS. The cells were resuspended in 200 μl of cold PBS, and 20 μl of an Rnase A (BestBio science, Shanghai, China) solution was added and incubated in a 37 °C water bath for 30 min. A 400-micron mesh screen was used for filtration. Then, 400 μl of propidium iodide (PI) (BestBio science, Shanghai, China) dye was added and the solution was gently mixed and incubated at 4 °C for 1 h in the dark. Analysis was performed using a flow cytometer (BD).

For apoptosis detection, cells were collected by centrifugation at 500×*g* at 4° C for 5 min. And washed twice with cold PBS. The cells mixed with 400 µl 1X Annexin V binding solution, and added with 5 µl Annexin V-FITC and incubated at 4° C in the dark for 15 min. PI was used to stain the nuclei. The labeling were detected using a flow cytometer. A small amount of cells in the suspension was smeared onto glass slides and observed under a fluorescence microscope.

### EdU cell proliferation assay

The cell density was adjusted to 4 × 10^4^ cells/ml. The cells were seeded in 24-well plates at 500 μl/well, and cultured in a 37 °C incubator with 5% CO_2_. After incubation overnight, the cells were infected with the virus. After 24 h of infection, the assay was carried out according to the instructions of an EdU kit (KeyGEN Biotech, Nanjing, China). The cells were incubated with an EdU solution for 2 h and then fixed with 4% paraformaldehyde. Triton X-100 (0.5%) was used to enhance the cell penetration, and a Click-It reaction was performed. The cells were stained with EdU and the nuclei were counterstained with Hoechst. The cells were examined using a fluorescent microscopy in the dark environment.

### Culture of primary glioma cells

We collected 2 cases of complete glioma surgical specimens, and carried out primary cell culture. After success, they were infected with adenovirus vectors to detect the effect of PCL2 on glioma cell proliferation. This experiment does not affect the patient's pathological diagnosis and has been approved by the ethics committee.

### Statistical methods

Statistical analysis was performed using SPSS 21.0 statistical software. The measurement data are expressed as the mean ± the SD, and each independent experiment was repeated 3 times. Multivariate mean comparisons were performed using one-way ANOVA. *P* < 0.05 was considered statistically significant.

## Conclusions

In summary, PCL2 plays a complex role in tumorigenesis. PCL2 can change the proliferation and decomposition of U87/U251 cells. As an important coenzyme of PRC2, PCL2 affects the expressions of core proteins EZH2 and EED, and changes the histone (H3K27, H3K9 and H3K4) methylation. The effect of EZH2 can be enhanced by increasing PCL2 expression, and this protein interaction is involved in changes in histone methylation. The overexpression of EZH2 may actually be an effect of some malignant tumors rather than the cause of some malignant tumors. At present, there is a preliminary understanding of the structure of PCL2 and its mechanism of action in U87/U251 cells. The overexpression of PCL2 is associated with tumor proliferation and progression. Further in-depth study of PCL2 will have an important impact on the diagnosis, treatment and prognosis of tumors.

## Electronic supplementary material

Below is the link to the electronic supplementary material.

Supplementary file1. PCL2 affects the number of nascent colonies of primary glioma cells (2019-37843). *P < 0.05, by one-way ANOVA. (TIF 62797 kb)

Supplementary file2. PCL2 affects the number of nascent colonies of primary glioma cells (2019-36563). *P < 0.05, by one-way ANOVA. (TIF 62797 kb)

## Abbreviations

PcG: Polycomb group
PRC1: Polycomb Repressive Complex 1
PRC2: Polycomb Repressive Complex 2
PCL2: Polycomb-like 2
MTF2: Metal regulatory transcription factor 2
EZH2: Enhancer of zeste 2 polycomb repressive complex 2 subunit
EED: Embryonic ectoderm development
SUZ12: Polycomb repressive complex 2 subunit
H3K27me3: Histone H3 lysine 27 trimethylation
ESC: Embryonic stem cell
LAML: Acute myeloid leukemia
MDM2: MDM2 proto-oncogene
RBBP7/RbAp46: RB binding protein 7, chromatin remodeling factor
RBBP4/RbAp48: RB binding protein 4, chromatin remodeling factor
DZNeP: 3-Deazaneplanocin A HCl
TCGA: The Cancer Genome Atlas
ACC: Adrenocortical carcinoma
KICH: Kidney chromophobe
LUAD: Lung adenocarcinoma
LUSC: Lung squamous cell carcinoma
OV: Ovarian serous cystadenocarcinoma
PRAD: Prostate adenocarcinoma
THCA: Thyroid carcinoma
DLBC: Diffuse large B-cell lymphoma
ESCA: Esophageal cancer
HNSC: Head and neck squamous cell carcinoma
GBM: Glioblastoma multiforme
LGG: Brain lower grade glioma
MOI: Virus multiplicity of infection
PHD: Plant homeodomain finger proteins
PTM: Post-translational modification
HMTase: Histone methyltransferase
DDR: DNA damage response
