# Supplementary figures and images for: Polycomb-like 2 regulates PRC2 components to affect proliferation in glioma cells
Source: J Neurooncol. 2020 May 21;148(2):259–71. doi: 10.1007/s11060-020-03538-0 (PMC7316845; doi:10.1007/s11060-020-03538-0)

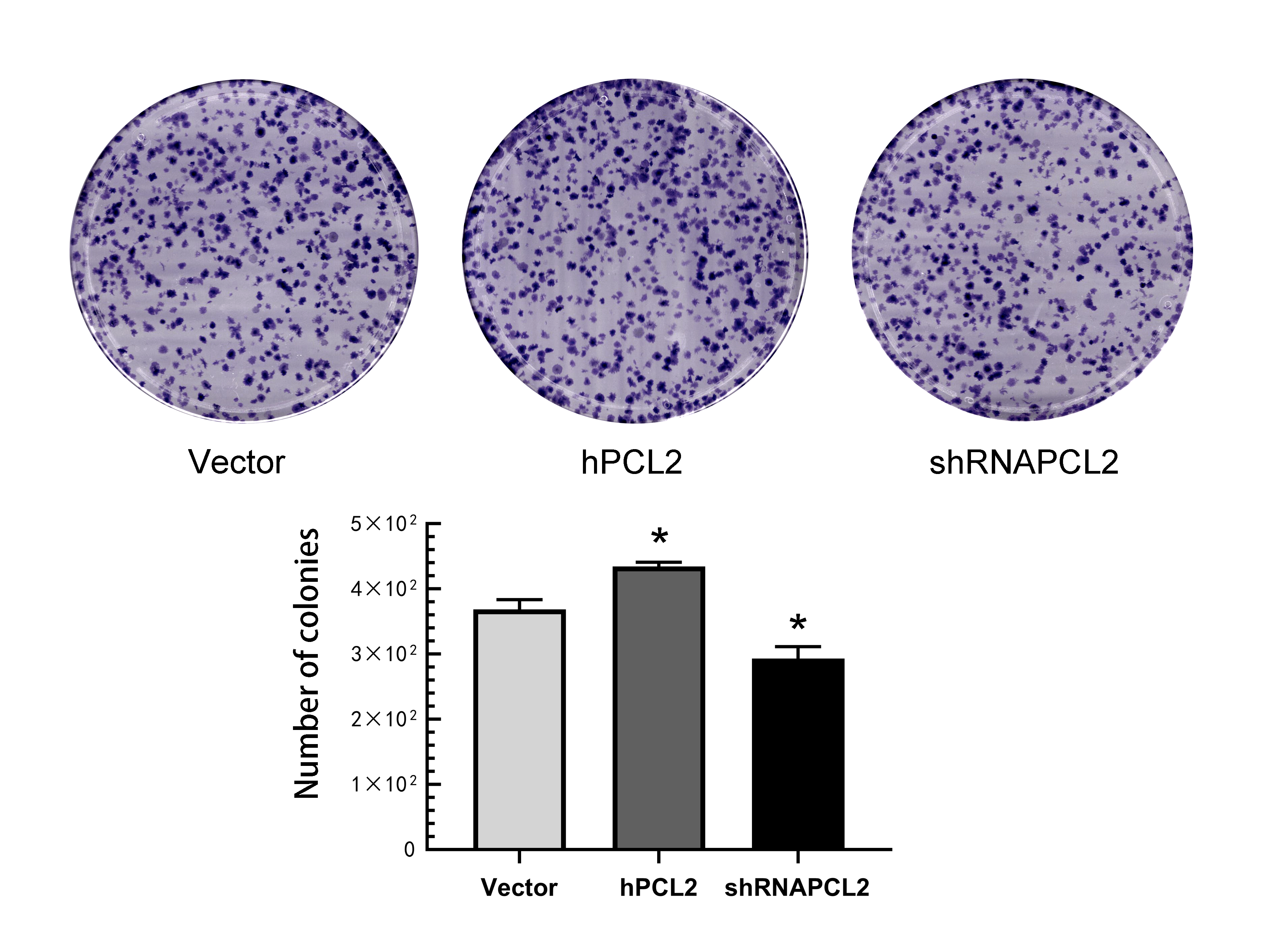

Supplement: Supplementary file 1 — Supplementary file1. PCL2 affects the number of nascent colonies of primary glioma cells (2019-37843). *P < 0.05, by one-way ANOVA. (TIF 62797 kb) [file 11060_2020_3538_MOESM1_ESM.tif]

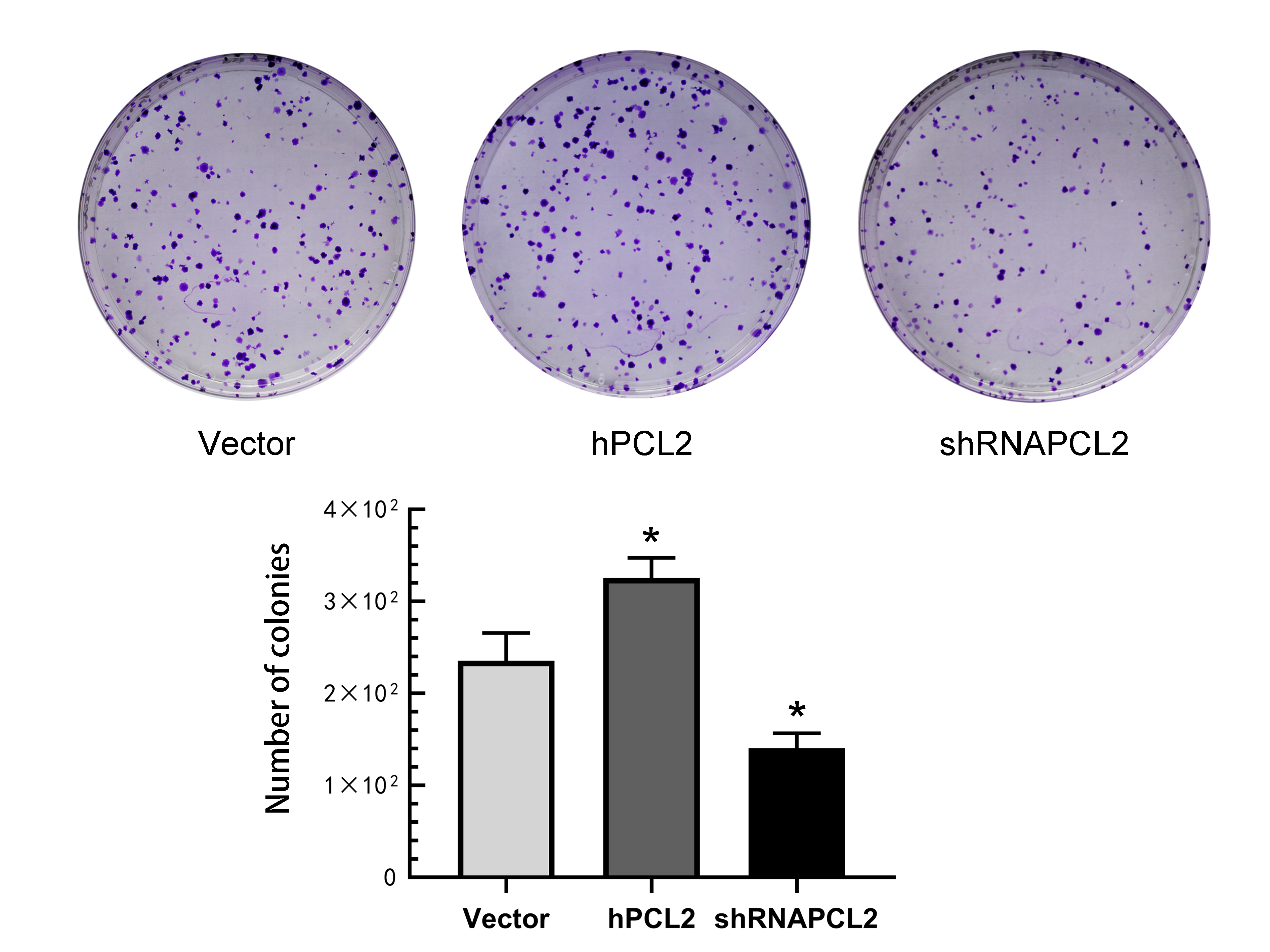

Supplement: Supplementary file 2 — Supplementary file2. PCL2 affects the number of nascent colonies of primary glioma cells (2019-36563). *P < 0.05, by one-way ANOVA. (TIF 62797 kb) [file 11060_2020_3538_MOESM2_ESM.tif]
